# Supplementary material for: A Pilot Evaluation of a School-Based Nutrition Education Program with Provision of Fruits and Vegetables to Improve Consumption Among School-Age Children in Palau
Source: Nutrients. 2025 Mar 12;17(6):994. doi: 10.3390/nu17060994 (PMC11944715; doi:10.3390/nu17060994)
Supplement: Supplementary file 1 [file nutrients-17-00994-s001.zip › nutrients-3447707-supplementary.pdf]

Table S1 Questionnaires on Nutritional Knowledge, Attitudes, and Behaviors Toward Vegetable and Fruit Consumption

category	Item	Question
**Nutritional Knowledge**		
Food category	K1	It is lunch time and Marty's lunch box contains the following: an apple, a carton of chocolate milk, yogurt, and grilled chicken. How many different food groups are in Marty's lunch box?
	K2	Which food does NOT belong in the grain group?
	K3	Which food does NOT belong in the vegetable group?
	K4	Which food does NOT belong in the fruit group?
	K5	Which food does NOT belong in the protein group?
	K6	Which food does NOT belong in the dairy group?
	K9	An example of a whole grain is:
Food servings	K7	How many total cups of fruit and vegetables combined should you eat each day?
	K8	How many cups should you have from the dairy group each day?
Choose health food	Which of the following would be a healthy choice? Check ALL that apply.	
	K10-1	(1) Fruit and yogurt
	K10-2	(2) Sports drink and cheese puffs
	K10-3	(3) Whole grain crackers and cheese
	K10-4	(4) Celery and peanut butter
	K11	Fruits is better than juice.
Health benefits of breakfast	Why is breakfast important? Check ALL that apply.	
	K12-1	(1) Helps you learn
	K12-2	(2) Gives you energy
	K12-3	(3) Helps you think and concentrate
**Attitudes Toward Vegetable and Fruit Consumption**		
	DA1	Vegetables are healthy for me
	DA2	Vegetables are important for me to eat
	DA3	I don't want to eat any vegetables
	DA4	Fruit is healthy for me
	DA5	Fruit is important for me to eat
	DA6	I don't want to eat any fruits
**Behaviors Toward Vegetable and Fruit Consumption**		
	DB1	I think vegetables taste good

DB2	I feel good when I eat vegetables
DB3	I like to eat vegetables
DB4	I think fruit taste good
DB5	I feel good when I eat fruit
DB6	I like to eat fruit

Abbreviation: DA, dietary attitude; DB, dietary behaviors.
